# Trellis Tone Modulation Multiple-Access for Peer Discovery in D2D Networks

**DOI:** 10.3390/s18041228

**Published:** 2018-04-17

**Authors:** Chiwoo Lim, Min Jang, Sang-Hyo Kim

**Affiliations:** 1 College of Information and Communication Engineering, Sungkyunkwan University, Suwon 16419, Korea; ichiwoo@skku.edu; 2 Samsung Electronics Co., Ltd., Suwon 16677, Korea; mn.jang@samsung.com

**Keywords:** trellis tone modulation multiple-access (TTMMA), device-to-device (D2D), discovery, non-orthogonal multiple-access (NOMA), multi-user detection

## Abstract

In this paper, a new non-orthogonal multiple-access scheme, trellis tone modulation multiple-access (TTMMA), is proposed for peer discovery of distributed device-to-device (D2D) communication. The range and capacity of discovery are important performance metrics in peer discovery. The proposed trellis tone modulation uses single-tone transmission and achieves a long discovery range due to its low Peak-to-Average Power Ratio (PAPR). The TTMMA also exploits non-orthogonal resource assignment to increase the discovery capacity. For the multi-user detection of superposed multiple-access signals, a message-passing algorithm with supplementary schemes are proposed. With TTMMA and its message-passing demodulation, approximately 1.5 times the number of devices are discovered compared to the conventional frequency division multiple-access (FDMA)-based discovery.

## 1. Introduction

Recently, the use of smartphones and tablet PCs has become common and mass multimedia communication has become more popular, resulting in a rapid increase in the amount of traffic passing through mobile communication networks. Since most of this mobile traffic is delivered over the core network, telecommunication service providers face serious network load problems. A good solution for coping with the large volume of traffic is thus required.

Device-to-device (D2D) communication is a distributed communication technology that can reduce the traffic load on the core network [1]. In the D2D communication, the traffic of an adjacent mobile station (MS) is delivered directly or only through a base station (BS) to which the two MSs belong. That is, the traffic is not delivered to the core network. D2D communication can solve the traffic overload problem by distributing concentrated traffic to the core network. Thus, D2D communications have attracted attention and have been actively studied in academia and industry [2,3,4,5,6,7,8,9,10]. Already, the 3rd Generation Partnership Project (3GPP) has been standardized as Proximity Services (ProSe) [11,12,13,14] and Sidelink [15] to include D2D communication functions. Qualcomm also has developed its own D2D communication technology called FlashLinQ [16].

The fifth-generation (5G) wireless communication systems aim to improve conventional technologies and provide stable and resource-efficient solutions according to various demands of the future. D2D communication has been proposed as an important technology to provide services including real-time data sharing [17] and it can be a part of 5G cellular network architecture for local offloading [18]. In addition, the D2D communication may play an important role when wireless sensor networks (WSNs) are integrated with 5G networks [19] and can be applied to various sensor network applications [20].

D2D communication can be controlled by a BS, but it can also be operated in a distributed manner when only a resource region is allocated by a BS [21]. On certain occasions, such as critical use in natural disasters, D2D communication needs to operate out-of-coverage [22,23]. In these cases, the D2D network becomes like a distributed network. In this paper, we discuss the distributed D2D operation rather than the BS controlled D2D operation.

One of most important features of D2D communication is peer discovery. Since the main advantage of D2D communication is the proximity service, a device needs to know the presence of other peers in its proximity. Peer discovery is a process used for identifying neighbor MSs, which can directly communicate with each other and are essential for constructing D2D communication. The general procedure for peer discovery of purely distributed networks is as follows. First, each MS broadcasts discovery message necessary for discovering the network address, interest, etc., and informs neighboring MSs of its existence. At the same time, each MS receives a signal broadcasted by the other MS and identifies the discovery message of the neighbor MS. In this way, the peer discovery constitutes a mesh network, as each MS transmits a discovery signal to all other MSs and receives discovery signals from all other MSs. In addition, since all MSs share a given resource for discovery, this can be regarded as a multiple-access process.

A conventional method of the peer discovery process is based on a combination of contention-based multiple-access and frequency division multiple-access [24,25]. All MSs in a network share a given set of resources and each MS transmits a discovery signal using only part of the resources. In the orthogonal frequency division multiplexing (OFDM) physical layer environment, resources are divided into time and frequency and each MS selects one of the time–frequency resources. This is a form of frequency division multiple-access. However, in the D2D peer discovery, there is no central controller such as a BS that allocates resources exclusively to each MS. Therefore, each MS uses various contention-based multiple-access techniques such as collision avoidance and collision detection to select the resources.

In the D2D communication in the distributed environment, if the density of a network increases, the orthogonal multiple-access (OMA)-based peer discovery method will not function well. In this situation, the performance can be improved by multi-user detection. The multi-user detection over a shared resource can be classified as a non-orthogonal multiple access (NOMA) technology. However, it is not straightforward to apply existing NOMA techniques to the discovery problem because those schemes assume that a distinct piece of information is allocated to each device and the receiver has the knowledge of the information. For example, in sparse code multiple access (SCMA), the receiver should know the used codebook sets for UEs that are participating in SCMA transmission and the codebooks should be distinct. However, it not easy to achieve this condition in distributed network environments. Similarly, in interleave-division multiple-access (IDMA), a different interleaver should be assigned to each UE and a receiver should know them for multi-user detection.

In this paper, we propose a new peer discovery framework based on trellis tone modulation multiple-access (TTMMA), which is applicable to a synchronized OFDM physical layer of 5G networks. Unlike the conventional method, TTMMA allows several MSs to modulate and transmit their signals on the same resource segment; therefore, TTMMA is indeed a non-orthogonal spectrum-sharing scheme. This mitigates the need for strict collision avoidance and collision detection techniques, in order to simplify the discovery procedure. In addition, the receivers efficiently separate and restore the superposed TTMMA modulated signals using message-passing demodulation. This message-passing demodulation increases the discovery capacity by supporting more MS signal recovery with the same resource usage. Finally, the TTMMA generates a single-tone discovery signal to concentrate the signal energy and fundamentally solves the problem of peak-to-average power ratio (PAPR) of OFDM. It thereby increases the transmission signal power and significantly improves the discovery range compared to existing technologies [24,25].

The remainder of this paper is structured as follows. In Section 2, we describe the operation of conventional FDMA-based discovery and investigate some of its problems. In Section 3, we show the procedure of TTMMA and then, in Section 4, the method of multi-user detection via message-passing algorithm is described. In Section 5, a performance comparison is performed between the proposed TTMMA and FDMA-based scheme. Finally, we conclude by discussing how the performance of TTMMA can be improved.

In this paper, both the conventional and proposed schemes are based on OFDM systems; therefore, the proposed scheme can be specified for most wireless systems with OFDM-based physical layer including LTE, future 5G, or Wi-Fi, and so on. In the following section, our is based on the 3GPP terms, so the BS is described as Evolved Node B (eNB) and the MS is described as User Equipment (UE).

## 2. System Model and Conventional FDMA-Based D2D Discovery

In this section, we briefly explain the system model for peer discovery in D2D networks and review the conventional FDMA-based discovery method that is based on contents of various papers and patents [24,25,26,27].

### 2.1. System Model

We assume that D2D networks are peer-to-peer ad hoc networks that operate in a distributed manner and no central coordinator exists. Every UE uniformly locates in a circular region with radius R. Each UE broadcasts a message through a discovery signal to its proximity. At the same time, each UE receives a signal sent by other UEs and identifies the discovery messages of the neighbor UEs. The resources for discovery are periodically allocated based on the OFDM physical layer. Synchronous transmission is assumed for efficient resource utilization as in the FlashLinQ [16]. The synchronization can be achieved in several ways [28].

In this D2D network, communications can be initiated by knowing the existence of proximity UEs. Therefore, each UE needs to discover as many peers as possible. To accomplish this, each UE tries to find all UEs around itself, but the goal is not always possible nor desirable. Depending on the discovery scheme, different numbers of UEs will be found within a given resource. The average number of discovered UEs is a major performance metric of the peer discovery in D2D networks. A good discovery scheme can achieve high desired discovery performance with low resource usage. 

In this paper, we evaluate the performance of the discovery scheme by using the observation UE at the center of the circular region, we let the uniformly distributed UEs transmit the discovery signal through the given discovery resource, and we observe the number of discovered UEs by detecting the observation UE.

### 2.2. FDMA-Based Discovery

**Figure 1:**
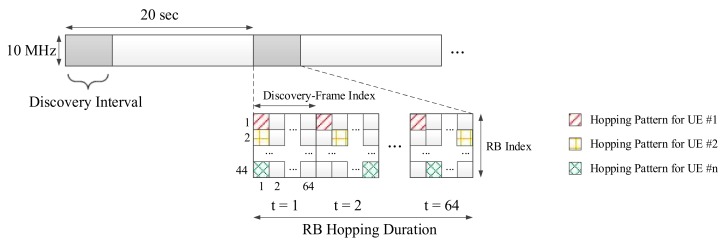
Timing diagram of repetitive LTE uplink frames including peer discovery time slot.

Figure 1 exhibits the timing diagram of repetitive LTE uplink frames with a peer discovery time slot [24]. This structure is based on the synchronized OFDM physical layer of the LTE uplink. The time interval for peer discovery is periodically allocated within the LTE uplink frame every 20 s. The UEs broadcast a discovery signal using time–frequency resources partially in this interval. Each UE can receive discovery signals from other UEs during a time when it does not transmit, due to the half-duplex nature of wireless communications.

Figure 1 also shows the OFDM resource map that constitutes the discovery interval. The basic unit, a single subcarrier of one OFDM symbol, is called a resource element (RE). The basic resource configuration unit of the LTE standard is called the resource block (RB), which consists of 12 subcarriers and 14 symbols, thus containing 168 REs. The number of RBs in the frequency domain in the discovery interval is determined by the frequency bandwidth. When 10 MHz bandwidth is used for discovery, 44 RBs are packed in the band. Since one discovery-frame spans 64 RBs in time, 44 × 64 RBs form a discovery-frame. Thus, one discovery interval has 64 discovery-frames.

Each UE selects one RB for each discovery-frame and transmits a discovery signal over the RB. The frequency domain has 44 parallel RBs when 10 MHz bandwidth is used for discovery. The UEs select one RB and transmit the discovery signals through an exclusively assigned resource. Therefore, this scheme can be regarded as frequency division multiple-access (FDMA) on the OFDM resource grid from the perspective of a device that aims to detect all of the discovery signals. In this case, each UE must prevent its own discovery signal from overlapping with signals of other UEs; therefore, the UEs select an RB using a collision avoidance technique. Collision avoidance is conducted as follows. (1) A UE that intends to join a D2D network listens to the discovery signals of other UEs without transmitting its own discovery signal. (2) The UE then calculates the energy level for all RBs in an entire discovery interval. (3) The UE randomly selects one of the RBs of the bottom 5% in measured energy and transmits the discovery signal over the selected RB in the next discovery interval [24], which we call the “minimum-energy-based selection rule”. This collision avoidance scheme approximately maximizes the distance between the UEs that use the same RB for discovery signal transmission.

Due to the half-duplex constraint, the UE cannot receive the discovery signals of other UEs while transmitting its own discovery signal. Therefore, as shown in Figure 1, the UE transmits the same discovery message through RBs changed by a predetermined hopping pattern over time in several discovery-frames. For example, this hopping pattern is determined by Latin-square [27].

Even if the UE uses the collision avoidance based on the minimum-energy-based selection rule, it cannot completely prevent proximate UEs from selecting the same RB because the collision avoidance is performed distributively. Therefore, each UE needs to monitor whether or not there is a nearby UE that uses the same RB. For this purpose, each UE listens to signals from other UEs in a discovery-frame without broadcasting a signal at a given time. If high energy is detected in the RB chosen by the UE, it is recognized as a collision and a resource is reselected by using a minimum-energy-based selection rule. This is a collision detection technique.

### 2.3. Drawbacks of FDMA-Based Discovery

The conventional FDMA-based discovery described in the preceding subsection is a combination of the frequency division multiple access and the contention-based multiple access. D2D discovery networks do not have a central scheduler such as an eNB, which exclusively allocates resources to UEs. Therefore, each UE must choose its own resource by itself.

The major problem of the FDMA-based discovery is that two or more UEs are not able to select the same RB or transmit signals. This causes two serious limitations:

(1) Complexity and delay of discovery process: The FDMA-based discovery does not allow collision; each UE performs the dynamic collision avoidance by listening to signals of other UEs and selects a vacant RB. This operation prevents a UE from transmitting a discovery signal immediately, thereby causing a delay. Even if each UE performs a collision avoidance operation, since it operates individually, collision can always occur and it must therefore perform the function of detecting a collision. If the discovery message recovery fails due to the collision, the reselection of a resource must follow, which again leads to a delay.
(2) Increased discovery overhead: In FDMA-based discovery, a multi-user detection (MUD) scheme is required when discovery signals of multiple UEs are collided on the same RB. To conduct the MUD, a channel code and successive interference cancellation (SIC) technique are required [26]. The use of a channel code reduces the resource efficiency because of the overhead of parity bits. In addition, SIC is required pilots for channel estimation and resources for collision detection, which increase the discovery overhead.

The above-mentioned problems need to be solved for more efficient D2D discovery. In this paper, we propose a new non-orthogonal multiple access using trellis tone modulation as a D2D discovery scheme. The proposed scheme enables the discovery to proceed smoothly even when multiple UEs participate in the discovery through the same resources.

## 3. Trellis Tone Modulation Multiple-Access

A new modulation scheme based on single-tone transmission is used for the new proposed multiple access scheme. We first consider a trellis composed of a number of states, where each state corresponds to a choice of a subcarrier, a tone. The trellis tone modulation is performed based on the tone index change between two consecutive OFDM symbols according to the discovery message, so that we can find its resemblance to the differential modulations. Each UE transmits its separately modulated signal, while signals from multiple UEs are superposed at the receiver. This section proposes trellis tone modulation multiple-access for peer discovery.

### 3.1. Overview of Trellis Tone Modulation

The modulation technique of TTMMA is introduced in this section. First, let $w=\left(w_{1},w_{2},\;\cdots ,\;w_{L}\right)$ be a binary discovery message of length *L*. The message includes the parity of cyclic redundancy check (CRC) codes. Each UE uses an OFDM resource grid that consists of M subcarriers and N symbols for the transmission of the discovery message, where the M×N resource grid is called a discovery resource unit (DRU). The discovery signal of UE U generated from w is then represented by a M×N matrix $X=\left[x_{1},\ldots ,x_{N}\right]$, where $x_{k}$ is the *κ*-th column vector of X. Each column of X indicates the frequency domain representation of the corresponding OFDM symbol generated from UE U. Since we use the single-tone transmission, each column of X has a Hamming weight of 1. The nonzero entries take the value of $\sqrt{P}$, where P is symbol power of transmission.

Let an N-tuple $t=\left(t_{1},\ldots ,t_{N}\right)$ be the sequence of tone index, that is corresponding to the index of the nonzero entry in each column of X. The signal X is determined if and only if t is determined. The message is fragmented into N parts as $w=\left(w_{1}:w_{2}:\ldots :w_{N}\right)$ where $w_{1}$ is a *b*_0_-tuple and others are *b*-tuples. The first tone index $t_{1}$ is determined by first *b*_0_ bits, $w_{1}$. The consecutive tone indices are determined by the recursive relation.

(1) $$t_{k}=f_{T}\left(t_{k-1},w_{k}\right),\;2\leq k\leq N$$

where $f_{T}:\left\{1,2,\ldots ,N\right\}\times {\left\{0,1\right\}}^{b}\to \left\{1,2,\ldots ,N\right\}$ is called the tone transition function. Note $N=\frac{L-b_{0}}{b}+1.$

Let us regard the tone indices as the states of the modulator. The tone transition function $f_{T}\left(\cdot \right)$ is fully characterized by a trellis diagram; therefore, we refer to our modulation scheme as a trellis tone modulation (TTM). We determine the trellis diagram by its incident matrix called the trellis matrix.

**Figure 2:**
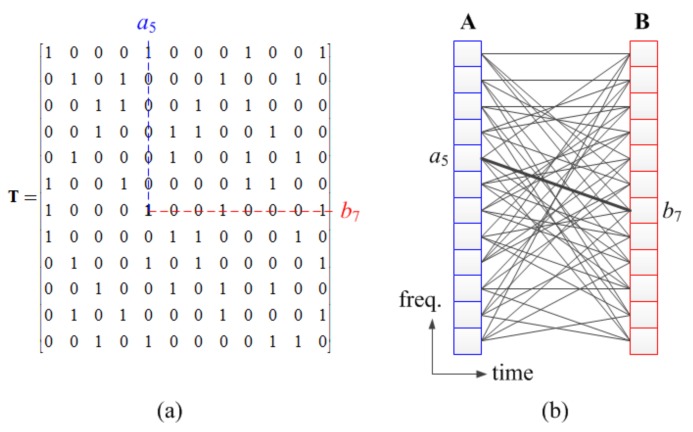
(**a**) Example of trellis matrix **T**; (**b**) Corresponding trellis diagram.

The trellis matrix $T=\left[t_{i,j}\right]\in {\left[0,1\right]}^{M\times M}$ is a binary matrix in which the number of rows and columns is equal to M. The number of 1s in each row and the number of 1s in each column are all equal to d and $d=2^{b}$ for a positive integer b. Ones in T indicate a possible transition from the state of the column index to the state of the row index. If $t_{p,q}=1$ and state q in the pre-state set and state p in the post-state set are then connected by an edge in the trellis diagram. The row and column weight d of T is referred to as the degree of the trellis diagram. Since the degree $d=2^{b}$, b bits can be encoded to the d state transitions via the corresponding edges, the trellis diagram can be regarded as a directional bipartite graph that we would call the “trellis graph”. Figure 2 exhibits an example of a trellis matrix and the corresponding trellis diagram, where M is 12 and d is 4.

The number of states M and the degree d of the trellis diagram are design parameters. The degree d is less than or equal to the number of states M. If d is larger, the trellis conveys more message bits and the transmission efficiency is increased. However, the demodulation complexity also increases. Therefore, a tradeoff occurs between the transmission or bandwidth efficiency and the demodulation complexity and the parameters are determined in order to meet the system requirements.

When constructing a trellis matrix using predetermined design parameters, the length of the cycle should be maximized as much as possible in order to improve demodulation performance. The design of the trellis matrix is similar to a parity-check matrix of the low-density parity-check (LDPC) code [29], where the number of states and the number of degrees are given. Therefore, the proposed methods for constructing the LDPC code can be used to construct the trellis matrix, for example, a progressive-edge-growth (PEG) algorithm [30]. If the number of states *M* is not large, the cycle of length 4 is necessarily included. Therefore, we do not need to put much effort into designing the trellis matrix.

### 3.2. Trellis Tone Modulation Procedure

In this subsection, we show the process of individual TTM. Assume that n transmitting UEs share the same DRU. Let $U^{\left(i\right)}$ be a UE among the n ones. Also, let $w^{\left(i\right)}=\left[w_{1}^{\left(i\right)},w_{2}^{\left(i\right)},\;\cdots ,\;w_{L}^{\left(i\right)}\right]$ be the discovery message of $U^{\left(i\right)}$. $w^{\left(i\right)}$ are fragmented into N parts as mentioned in previous subsection. The trellis matrix is designed for the given discovery resource unit, a single trellis matrix **T** is used for all n UEs.

**Figure 3:**
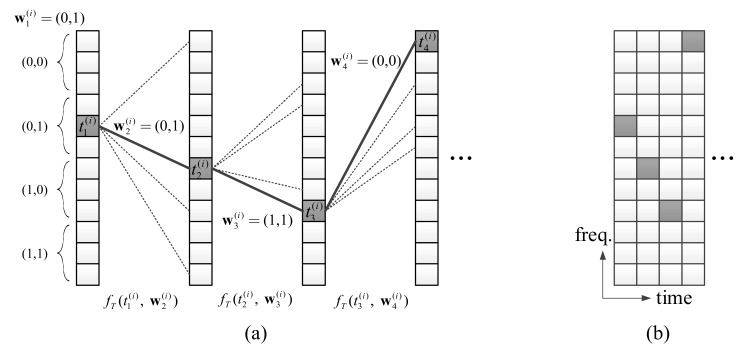
(**a**) Example of trellis tone modulation generated by referring to the trellis diagram in Figure 2; (**b**) Corresponding trellis tone modulation signal in orthogonal frequency division multiplexing (OFDM) resource grid.

The tone index of the first symbol, $t_{1}^{\left(i\right)}\;$ is determined by $b_{0}$ bits of $w_{1}^{\left(i\right)}$. Since the number of tones is M in each symbol, a choice of single-tone can represent the maximum $\left\lfloor \log_{2}M\right\rfloor$ bits. Thus, $b_{0}$-bits, $b_{0}\leq \lfloor \log_{2}M\rfloor$ can be represented by the tone selection in the first symbol. For simplicity, we assume $2^{b_{0}}|M$. The tone index set is partitioned into $2^{b_{0}}$ groups and the choice of a group can represent $b_{0}$ bits. Single tone selection is conducted uniform-randomly within the group determined by the message bits. Figure 3a shows an example of the tone selection where $b_{0}$ is 2 and M is 12. If the $w_{1}^{\left(i\right)}$ is (0,1), one of the 4-th, 5-th and 6-th tones can be randomly selected. In the example of Figure 3a, the selected tone is the 5-th tone, so $t_{1}^{\left(i\right)}=5.$

The tone indices of following symbols are determined by the previous tone index and the tone transition function. The tone index $t_{k+1}^{\left(i\right)}$ of the (*k* + 1)-th symbol is determined by $t_{k}^{\left(i\right)}$, $w_{k+1}^{\left(i\right)}$ and the tone transition function $f_{T}\left(‧\right)$, for k=1, …, N−1. For example, if $t_{1}^{\left(i\right)}$ = 5 and $w_{2}^{\left(i\right)}=\left(0,1\right)$, $t_{2}^{\left(i\right)}$= $f_{T}\left(5,w_{2}^{\left(i\right)}\right)$ = 7 as shown in Figure 3a.

Figure 3a gives a more comprehensive illustration of the entire TTM process. If the discovery message intended to be transmitted by the UE is (0,1,0,1,1,1,0,0,...), the single-tone of the first symbol is determined as the fifth tone, which is one of the tones belong to the region corresponding to the first 2 bits (0,1), as described above. The single-tone of the second and subsequent symbols is determined according to the single-tone of the previous symbol and two bits message, respectively. Algorithm 1 summarizes the procedure of TTM for a UE.
**Algorithm 1** Trellis Tone Modulation Procedure**Require**: Message vector $w=\left(w_{1}:w_{2}:\ldots :w_{N}\right)$, trellis matrix **T** Determine $t_{1}^{\left(i\right)}$ with $w_{1}^{\left(i\right)}$ and $x_{1}^{\left(i\right)}\leftarrow t_{1}^{\left(i\right)}$
Note $x_{1}^{\left(i\right)}$ is the column vector the $t_{1}^{\left(i\right)}$-th element having only the value of $\sqrt{P}$ and the other elements having a value of zero. **for**
*k* = 1 to *N* − 1 **do** $t_{k+1}^{\left(i\right)}$ = $f_{T}\left(t_{k}^{\left(i\right)},w_{k+1}^{\left(i\right)}\right)$, $x_{k+1}^{\left(i\right)}\leftarrow t_{k+1}^{\left(i\right)}$
 **end for** $X^{\left(i\right)}\leftarrow \left[x_{1}^{\left(i\right)},\ldots ,x_{N}^{\left(i\right)}\right]$

For use in the next section, we derive a relation over the OFDM signal domain, which is equivalent to (1). The tone-transition matrix $H_{k}^{\left(i\right)}$, which represents the form in which the tone transits from the previous *k*-th symbol to the (*k* + 1)-th symbol of the $U^{\left(i\right)}$, is defined as a matrix that has an element in the $\left(t_{k}^{\left(i\right)},\;t_{k+1}^{\left(i\right)}\right)$ position in the trellis matrix **T** and all other elements are zero. Therefore, $H_{k}^{\left(i\right)}$ has only one nonzero element. In particular, $H_{k}^{\left(i\right)}$ is a matrix with only one element 1, because the trellis matrix **T** is limited to a simple binary matrix with only 0 and 1 elements. Thus, we have

(2) $$x_{k+1}^{\left(i\right)}=H_{k}^{\left(i\right)}x_{k}^{\left(i\right)}$$

Using (2), we can obtain the all $x_{k+1}^{\left(i\right)}$ for k=1, …, N−1 and then makes the discovery signal $X^{\left(i\right)}$ for $U^{\left(i\right)},$ as shown in Figure 3b.

### 3.3. Multiple Access of Trellis Tone Modulation Signals

**Figure 4:**
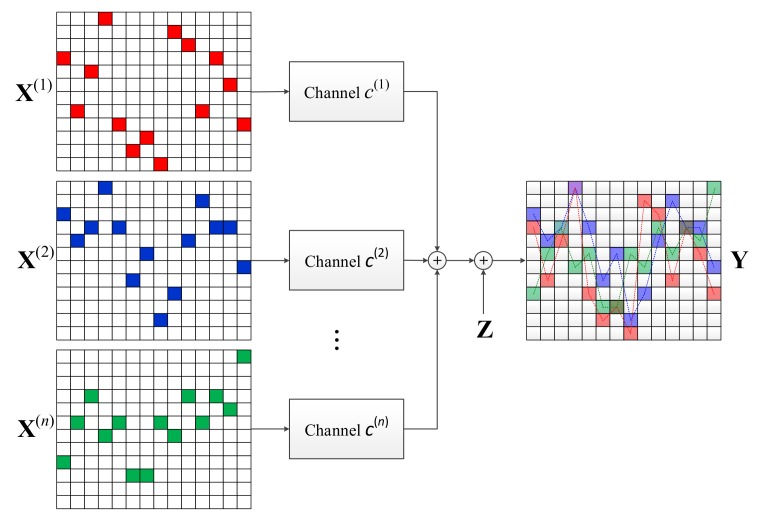
Conceptual description of multiple-access of trellis tone modulation (TTM) signals.

We now consider the multiple access scenario where a receiving UE attempts to detect the messages sent from multiple UEs via a DRU. As shown in Figure 4, $U^{\left(i\right)}$ performs the TTM of the discovery message and constructs $X^{\left(i\right)}$.

Let the channel gain from $U^{\left(i\right)}$ to the receiver be $c^{\left(i\right)}$. We assume the channel gain is a value considering the channel environments such as path-loss, fading, etc., As described above, the trellis width *M* is determined such that the entire DRU passes through a flat fading channel. We also consider the channel is static in time over a single DRU. Of course, if *N* is large, the channel can change in a DRU under a time varying channel. However, to ensure the valid operation of the message-passing demodulation, which is introduced in the next section, the quasi-static fading channel is assumed. 

The received signal **Y** is a superposition of transmitted signals weighed by channel constants,

(3) $$Y=\sum_{i=1}^{n}c^{\left(i\right)}X^{\left(i\right)}+Z$$

where $Z\in {\mathbb{C}}^{M\times N}$ is the independent additive complex Gaussian noise with variance $\sigma^{2}$ for each entry. Figure 4 shows how **Y** is obtained. The detection of the multiple messages from this superposed signal is addressed in the next section.

The signal-to-noise ratio (SNR) is assumed to be large even if the signal undergoes path loss, since each UE transmits the signal with sufficiently large power through single-tone signal transmission. The network is therefore interference-dominant.

## 4. Multi-User Detection of TTMMA via Message-Passing

In this section, we describe the process of multi-user detection via a message-passing algorithm. This multi-user detection algorithm is effective in both single user and multiple user scenarios. In addition, we propose some supplementary techniques such as tone-space expansion, pre-prediction, etc., for efficient realization of the message-passing-based multi-user detection.

At the transmitter, $w_{1}^{\left(i\right)}$ is represented only by $x_{1}^{\left(i\right)}$ and then, $x_{k+1}^{\left(i\right)}$ is generated only depending on $w_{k+1}^{\left(i\right)}$ and $x_{k}^{\left(i\right)}$ for k≥2. Since the modulation of TTMMA discovery signal is Markovian, $w_{k+1}^{\left(i\right)}$ can separately be determined by the tone transition from $x_{k}^{\left(i\right)}$ to $x_{k+1}^{\left(i\right)}$. Therefore, the receiver carries out multi-user detection by using only $y_{k}$ and $y_{k+1}$ to find $w_{k}^{\left(i\right)}$ for each k, where $y_{k}$ is the *k*-th column vector of Y.

Now let us focus on the multi-user detection procedure between $y_{k}$ and $y_{k+1}$ to find $w_{k}^{\left(i\right)}$ for all i. From (3), $y_{k}$ is broken down as

(4) $$y_{k}=\sum_{i=1}^{n}c^{\left(i\right)}x_{k}^{\left(i\right)}+z_{k}={\tilde{x}}_{k}+z_{k}$$

where ${\tilde{x}}_{k}=\sum_{i=1}^{n}c^{\left(i\right)}x_{k}^{\left(i\right)}$ and $z_{k}\in {\mathbb{C}}^{M}$ is the corresponding noise vector and $y_{k+1}$ is given by

(5) $$y_{k+1}=\sum_{i=1}^{n}c^{\left(i\right)}x_{k+1}^{\left(i\right)}+z_{k+1}=\sum_{i=1}^{n}c^{\left(i\right)}(H_{k}^{\left(i\right)}x_{k}^{\left(i\right)})+z_{k+1}=\sum_{i=1}^{n}H_{k}^{\left(i\right)}\left(c^{\left(i\right)}x_{k}^{\left(i\right)}\right)+z_{k+1}$$

Then we derive the optimal joint detection problem given by

(6) $${\left\{{\hat{H}}_{k}^{\left(i\right)}\right\}}_{i=1}^{n}=\arg\underset{A_{i}}{\min}\|y_{k+1}-\sum_{i=1}^{n}A_{i}\left(c^{\left(i\right)}x_{k}^{\left(i\right)}\right)+z_{k+1}\|$$

where $A_{i}$ is a possible form of $H_{k}^{\left(i\right)}$. There is only one non-zero element 1 in $H_{k}^{\left(i\right)}$ and the number of possible locations of the non-zero element is dM. Thus, the joint ML of (5) requires ${\left(dM\right)}^{n}$ comparisons and even n is unknown to the receiver. Thus, it is impractical to be exploited. Therefore, using the sparsity of $H_{k}^{\left(i\right)}$ and $x_{k}^{\left(i\right)}$, we modify (5) into an easy-to-solve form, that is given by

(7) $$y_{k+1}=H_{k}\left(\sum_{i=1}^{n}c^{\left(i\right)}x_{k}^{\left(i\right)}\right)+z_{k+1}=H_{k}{\tilde{x}}_{k}+z_{k+1}$$

where $H_{k}\in {\mathbb{R}}^{M\times M}$ is a newly defined multiple-access tone-transition matrix. Since trellis tone modulated signals of multiple UEs are superposed and separated across the symbols, $H_{k}$ is no longer a binary matrix, but a real matrix. The number of non-zero elements is less than or equal to n and the possible locations of non-zero elements in $H_{k}$ is still restricted by T. Now the multi-user detection problem is to find $H_{k}$ instead of finding $H_{k}^{\left(i\right)}$ for all i. After obtaining $H_{k}$, we decompose $H_{k}$ into $H_{k}^{\left(i\right)}$ by applying a combinatorial method to $H_{k}$ for all k.

From (4) and (7),

(8) $$y_{k+1}=H_{k}\left(y_{k}-z_{k}\right)+z_{k+1}$$

The problem in (8) is different from the conventional one of linear equations. While $y_{k}$ is the desired unknown vector with given $y_{k+1}$ and $H_{k}$ in the conventional linear equation problems, $H_{k}$ is the desired unknown matrix with given tow vectors $y_{k}$ and $y_{k+1}$ in (8). The number of unknown variables is dM and we have M individual equations, so it is an underdetermined problem. Thus, it is hard to solve the problem in (8), but we mitigate the problem by using the fact that $H_{k}$ is sparse and the possible locations of non-zero elements in $H_{k}$ are known by the trellis matrix T. In that sense, the message-passing is one of the most effective ways to solve such a problem with practical computational complexity [31]. In the next subsection, we propose a message-passing method to solve the multi-user detection problem in (8).

### 4.1. Message-Passing Algorithm

**Figure 5:**
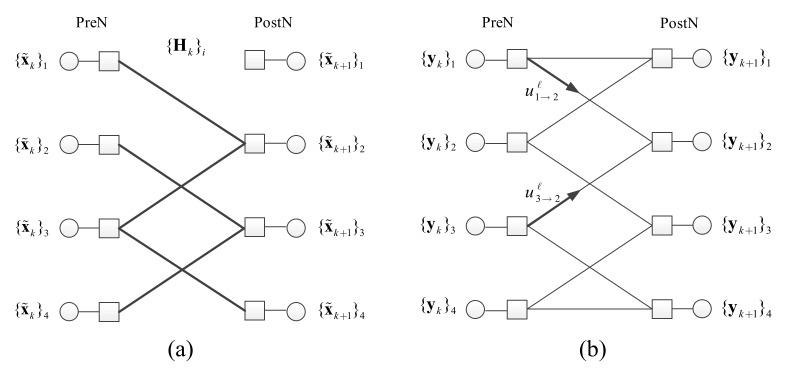
(**a**) Example of multiple-access tone transition graph; (**b**) Example of base graph corresponding to an example T.

The first goal of the message-passing algorithm is to determine $H_{k}.$ The linear equation (8) with a sparse matrix $H_{k}$ can be represented by a bipartite graph as given in Figure 5a. The bipartite graph, which we call the multiple-access tone transition graph, is composed of pre-state nodes (PreN) and post-state nodes (PostN) onto which ${\tilde{x}}_{k}$ and ${\tilde{x}}_{k+1}$ are loaded respectively. Let ${\left\{A\right\}}_{j}$ be the *j*-th row vector of matrix A and ${\left\{A\right\}}_{i,j}$ be the entry at the i-th row and the j-th column. The edge connecting PreN *i* and PostN *j* is weighted by ${\left\{H_{k}\right\}}_{i,j}$. If forward messages from left to right are generated such that the node value is multiplied to edge weight, then in the i-th right node the sum of incoming messages are equal to the node value, equivalently,

(10) $${\left\{{\tilde{x}}_{k+1}\right\}}_{i}={\left\{H_{k}\right\}}_{i}{\tilde{x}}_{k}$$

Finding $H_{k}$ is equivalent to determination of the graph structure.

We achieve the goal by validating and pruning edges from a base graph via message-passing decoding with the received signals $y_{k}$ and $y_{k+1}$. First, the base graph is defined as a graph with the same state node sets where every possible state transition due to T is represented as an edge. Naturally, the multiple-access tone transition graph is a subgraph of this base graph. The base graph corresponding to an example

$$T=\left[\begin{array}{c} \begin{array}{c} 1\\ 1 \end{array}\begin{array}{c} \;1\\ \;0 \end{array}\begin{array}{c} \;0\\ \;1 \end{array}\begin{array}{c} \;0\\ \;0 \end{array}\\ \begin{array}{c} 0\\ 0 \end{array}\begin{array}{c} \;1\\ \;0 \end{array}\begin{array}{c} \;0\\ \;1 \end{array}\begin{array}{c} \;1\\ \;1 \end{array} \end{array}\right]$$

is given in Figure 5b. When decoding is conducted, first the nodes are initialized with $y_{k}$ and $y_{k+1}$ and then messages are exchanged between PreNs and PostNs through the edges. Edges in the base graph can be validated or pruned during the demodulation; the graph is then reduced by the node processing. The node processing is basically the validation (or pruning) of edge candidates. This is equivalent to find the non-zero elements in ${\left\{H_{k}\right\}}_{i}$ for the node. The PostN check if there is a possible edge connection pattern that satisfies the matching condition; the incoming messages are well matched to the node value ${\left\{y_{k+1}\right\}}_{i}$ with respect to the edge combination. If an edge connection pattern is confirmed then some edges are validated and some edges are pruned and outgoing messages are generated based on the decision. The messages for pruned edges will be ignored or nulled whereas the messages for validated edges are set as the incoming message. For non-confirmed nodes, the outgoing message is determined by the subtraction of the sum of incoming messages from other nodes from the node value ${\left\{y_{k+1}\right\}}_{i}.$

The backward message-passing is exactly the same as the forward processing other than the fact that the relation is inverted as

(11) $${\left\{{\tilde{x}}_{k}\right\}}_{i}={\left\{H_{k}^{-1}\right\}}_{i}{\tilde{x}}_{k+1}$$

Iterative processing of the forward and backward message-passing gives a reliable detection of multiple UE messages.

When the node processing is conducted at PostN q, there are $2^{d}$ edge combinations where d is the degree of PostN q. Let p be a binary vector of length d and indicate the edge connection pattern where zero indicates the edge pruned and $u_{q}$ be the vector of incoming messages to PostN q. Let $C_{p}=u_{q}p^{T}$ be the combined message with respect to p. Then $C_{p}$ is compared with the node value ${\left\{y_{k+1}\right\}}_{q}$. If they are sufficiently close as ${\left\|\{y_{k+1}\right\}}_{q}-C_{p}{\|}^{2}\leq \left(w_{H}\left(p\right)+1\right)\delta$, where δ is the noise power and $w_{H}\left(\cdot \right)$ denotes the Hamming weight, we confirm the edge connection pattern p is valid. The edges are validated or pruned according to the confirmed pattern p. Since one noise component is added to the value of node and the message, $w_{H}\left(p\right)+1$ noise components is included in ${\left\{y_{k+1}\right\}}_{q}-C_{p}$. If ${\left\{y_{k+1}\right\}}_{q}$ satisfies the pattern p, then only $w_{H}\left(p\right)+1$ noise components remain in ${\left\{y_{k+1}\right\}}_{q}-C_{p}$, so this value follows the complex Gaussian distribution $CN\left(0,\left(w_{H}\left(p\right)+1\right)\sigma^{2}\right)$. Therefore, when α is a constant larger than 1, it is set to $\delta =\alpha \sigma^{2}$ and the process of checking ${\left\|\{y_{k+1}\right\}}_{q}-C_{p}{\|}^{2}\leq \left(w_{H}\left(p\right)+1\right)\delta$ is used to determine whether the remaining signal component of ${\left\{y_{k+1}\right\}}_{q}-C_{p}$ is noise only. The details of the message-passing procedure is given in the following.

(1) The forward message $u_{p\to q}^{l}\;$ is passed from the p–th PreN to the q–th PostN in the *l*-th iterative message-passing. Similarly, $v_{q\to p}^{l}$ is the backward message passed from the q–th PostN to the p–th PreN. The initial forward messages are set as $u_{p\to q}^{1}={\left\{y_{k}\right\}}_{p}$.
(2) In the *l*-th iterative message-passing, the q–th PostN compares its value ${\left\{y_{k+1}\right\}}_{q}$ with all candidate patterns $C_{p}=u_{q}p^{T}.$ The condition ${\left\|\{y_{k+1}\right\}}_{q}-C_{p}{\|}^{2}\leq \left(w_{H}\left(p\right)+1\right)\delta$ is checked for all **p**. For example, in Figure 5b, the second PostN compares the combined messages $C_{\left(00\right)}=0$, $C_{\left(10\right)}=u_{1\to 2}^{l}$, $C_{\left(01\right)}=u_{3\to 2}^{l}$ and $C_{\left(11\right)}=u_{1\to 2}^{l}+u_{3\to 2}^{l}$ with ${\left\{y_{k+1}\right\}}_{2}$.
(3) When at least one case satisfies the above matching condition, the q–th PostN determines the pattern that minimizes ${\left\{y_{k+1}\right\}}_{q}-C_{p}$. The q–th PostN then passes the value corresponding to determined pattern p to the neighbor PreNs. For validated edges, the outgoing message is set to the incoming edge. For pruned edges whose corresponding entry in p is zero, no message is passed. If no pattern is confirmed, the differential message $\sum_{v\in V_{q}\backslash \left\{p\right\}}u_{v\to q}^{l}$, where $V_{q}$ is the set of neighbors of q, is generated and sent to each individual neighbor PreN p. For example, in Figure 5b, the second PostN does not find a satisfying candidate, passes the ${\left\{y_{k+1}\right\}}_{2}-u_{3\to 2}^{l}$ value to the first PreN and the ${\left\{y_{k+1}\right\}}_{2}-u_{1\to 2}^{l}$ value to the third PreN.
(4) In the (*l* + 1)-th iterative message-passing process (l≥1), the PreNs and the PostNs are performed in the same manner. A threshold-based check is performed on all candidates and if a satisfactory candidate is found, it is determined and passed. If a satisfactory candidate is not found, a differential message is generated and passed.
(5) If all PreNs and PostNs are satisfied, or the number of iteration for message-passing reaches a predetermined maximum number, the demodulation is terminated.

If the message-passing between the *k*-th symbol and the (*k* + 1)-th symbol is performed, a reduced trellis graph is obtained. Candidate codewords can be obtained by concatenating the trellis graphs for all k, 1≤k≤N−1, if a connected path exists and can be separated then the tone-path may yield a valid codeword. The verification can be performed by using embedded CRC codes. In the proposed message-passing decoding, the computational complexity (in total number of additions) can be given by

(12) $$\left(N-1\right)\times 2I_{max}\times M\times \left(\sum_{i=1}^{d}\left(\begin{array}{c} d\\ i \end{array}\right)\left(i-1\right)+2^{d}\right)$$

where $I_{\max}$ denotes the maximum number of iterations. In (12), the sum $\sum_{i=1}^{d}\left(\begin{array}{c} d\\ i \end{array}\right)\left(i-1\right)$ is the number of additions for generating $C_{p}=u_{q}p^{T}$ in each node and $2^{d}$ represents the number of comparisons in ${\left\|\{y_{k+1}\right\}}_{q}-C_{p}{\|}^{2}\leq \left(w_{H}\left(p\right)+1\right)\delta$ for all **p**. The complexity of the comparison is equivalent to that of the addition. Note that the complexity in (12) is an upper bound, which can be reduced by optimization. The message-passing is performed between PostNs and PreNs only for $I_{\max}$ iterations (e.g., $I_{\max}=3)$, which keeps the complexity of the demodulation scheme sufficiently low. Sometimes it is not straightforward to distinguish tone-paths from the concatenated trellis graphs simply. That problem is addressed in the next subsection.

### 4.2. Tone-Space Expansion

**Figure 6:**
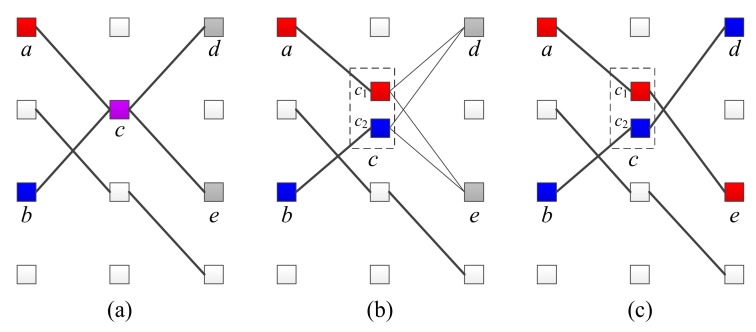
(**a**) Example of merger and separation occurrence of the tone-path; (**b**) Tone-space expansion with merged paths; (**c**) Performing message-passing and routing according to tone-space expansion.

Suppose that the tone-paths of two UEs are merged at a certain node and separated in the next step, as shown in Figure 6a. In this case, four tone-paths should be taken account of in the receiving UE, as shown in (*a*-*c*-*d*), (*a*-*c*-*e*), (*b*-*c*-*d*) and (*b*-*c*-*e*). The number of candidate codewords increases exponentially as a separation occurs after a merger, which greatly affects the demodulation complexity.

We use a method called tone-space expansion in order to reduce the demodulation complexity. When two or more tone-paths overlap, the message-passing process can detect the values are superposed at each position. Therefore, by separating overlapping symbols and running a message-passing process on the next symbol, it is possible to prevent an increase in the number of candidate codewords due to the separation. For example, if the values at *a* and *b* of the (*k* − 1)-th symbol are superposed on the *k*-th symbol *c* as shown in Figure 6b, they are separated into *c*_1_ and *c*_2_, respectively. Then, we connect *c*_1_ and *c*_2_ to the (*k* + 1)-th symbol, similar to the existing *c*, and perform the next message-passing. In other words, the tone-space expansion method adjusts the trellis diagram when superposition is checked during message-passing.

The tone-space expansion is very simple, but greatly reduces the demodulation complexity and improves performance. As shown in Figure 6c, the tone-space expansion can be used to distinguish the tone-path, even if separation occurs after superposition, so that the number of candidate codewords does not increase. Tone-space expansion also improves demodulation performance by decomposing the complex-path, which is two or more tone-transitions in which superposition and separation occur at the same time.

### 4.3. Pre-Prediction Method

In the first stage of demodulation, the first tones of the tone-paths should be detected. However, the tone-space expansion cannot apply to the superposed tones in the first symbol. So, we propose the pre-prediction method for demodulation of the first tone.

In order to perform the pre-prediction, the receiving UE copies the M×N receiving trellis matrix **Y** and inverts the order of the columns so that the order of the columns is inverted to form the reverse-direction trellis matrix [**y***_N_*, **y***_N_*_-1_,…, **y**_1_]. This reverse-direction trellis matrix is then connected to the existing received trellis matrix **Y** to form an expanded trellis matrix [**y***_N_*, **y***_N_*_-1_,…, **y**_2_, **y**_1_, **y**_2_,…, **y***_N_*]. When performing messages-passing for the opposite part of the extended trellis matrix [**y***_N_*, **y***_N_*_-1_,…, **y**_1_], the existing trellis diagram **T** uses the graph shown in the opposite direction. Through the pre-prediction, it is possible to confirm whether the first symbol is overlapped and thus the tone-space can be expanded.

### 4.4. Successive Interference Cancellation and Threshold Adjustment

The performance can be improved by performing successive interference cancellations (SICs) in the demodulation process of the TTMMA signal. If only part of the tone-path of all *n* UEs is recovered, removing the signal from the received signal reduces the number of signals remaining in the corresponding DRU, so that additional signal detection can be expected when the demodulation is performed again through the message-passing demodulation.

Channel estimation is required for the successive interference cancellation operation. The TTMMA does not use a separate pilot for channel estimation because the channel gain can be directly detected from the single-tones. When performing message-passing with tone-space expansion, the demodulator can determine whether or not the detected tone-path is superposed. If a tone-path is determined to be a single path or a separate path in the *k*-th symbol, the signal value at the corresponding position in the *k*-th symbol can be used as the channel estimation value. If the channel gain varies over time over *N* symbols, the channel estimation for the superposed symbol may be performed using an average value of the signals in adjacent symbols determined as single-tones, or a weighted-sum can be used.

In addition, the receiving UE can further demodulate by adjusting the threshold even if it has not detected any tone-path in the demodulation process. Since the background noise is a random variable, the noise added to a particular symbol may have an unusually large value compared to the average value. Therefore, if the receiving UE does not identify any discovery signal, it can attempt to demodulate again by adjusting the default threshold value δ used in the message-passing process. For example, if the threshold value $\delta =2\sigma^{2}$ has been set for the first message-passing process, it is increased to $\delta =3\sigma^{2}$, $\delta =4\sigma^{2}$, etc. in the next message-passing process. In this way, it is possible to expect additional tone-path discrimination by alleviating the condition of the tone-transition test through upward adjustment of the threshold value.

Algorithm 2 summarizes the procedure of multi-user detection of TTMMA signals.


**Algorithm 2 Procedure of Multi-User Detection of TTMMA Signals**
Receive **Y** Perform Pre-Prediction **for**
k=1 to N−1
**do** Find $H_{k}$ using message-passing **end**
Concatenate $H_{k}$’s Extract valid tone-paths (or codeword) with codeword verification using CRC code. Tone-space expansion is used for discriminating SIC or Threshold Adjustment (if needed) and Return to Perform Pre-Prediction step.

## 5. Performance Evaluation

This section compares and evaluates the discovery performance of the conventional FDMA-based scheme and the TTMMA scheme based on the 3GPP LTE uplink system. Their computational complexities are also compared. We consider a situation where a total of *n* transmitting UEs transmit a discovery signal using given DRUs in a circular network with a radius of 500 m, which is the set-up for convenience of evaluation. Each transmitting UE broadcasts 150 bits of discovery message, which includes 16 bits of CRC code for error detection. The positions of transmitting UEs are determined uniformly at random in the given region and the receiving UE is located at the origin. Table 1 shows the simulation assumptions in detail.

As shown in Table 1, we use the D2D outdoor-to-outdoor model [15] as propagation model and two channel models. One channel model is Block Rayleigh fading and another channel model is TDL-A [32]. Even though channel models in [32] are designed for above 6 GHz carrier frequency, these channel models are also generally considered for all evaluations in 3GPP standards including below 6 GHz carrier frequency. Especially, TDL-A 30 ns is the most common channel model considered for 5G link level simulations such as Ultra-Reliable Low Latency Communication (URLLC) and NOMA.

In the FDMA-based scheme, a UE transmits a discovery signal using a DRU1 that consists of 12 subcarriers and 14 symbols. One symbol of DRU1 is used for transmission–reception switching and another one symbol of DRU1 is used for Demodulation Reference Signal (DMRS). Therefore, each UE can use 12 × 12 = 144 REs. Each UE encodes a 150 bits message using polar codes [33], which shows the best performance in short-length and adopted in the 5G standardization, to produce a 288 bits codeword, and modulates the 288 bits codeword into 144 QPSK symbols, which are then loaded onto 144 REs. At this time, the transmission power is evenly distributed to the 12 subcarriers. The DMRS for channel estimation are generated by a Hadamard sequence of length 8. That is, the collision probability is 1/8. The interference in pilots are fully interfered if the sequence is collided with 1/8 probability, and are partially interfered with 1/8 power if the sequence is not collided with 7/8 probability. Since two or more UEs can use the same DRU, the receiving UE performs channel estimation and a general SIC operation accordingly. We assume the ideal channel estimation.

In the TTMMA scheme, each UE uses a DRU2 that consists of 12 subcarriers and 76 symbols. Since one symbol is used for transmission-to-reception switching in the same way as the FDMA-based scheme, each UE generates a TTMMA signal on 12 × 75 REs. Because the transmit power is focused on one tone per symbol, single-tone transmission gain of 10.7 dB and the PAPR gain of 6 dB is considered in this simulation. The 150 bits of the discovery message are mapped to 2 bits from the first symbol to a single-tone position and 148 bits to a tone-transition between 75 symbols. No pilots or collision detection techniques are used. The receiving UE performs demodulation using a message-passing process by adding the tone-space expansion and the pre-prediction. In the message-passing process, the maximum iteration number is fixed at 3. Even if only one candidate codeword is not found, the receiving UE increases the threshold value two-fold and further attempts demodulation up to three times. Unlike the FDMA-based scheme, the receiving UE doesn’t performs the SIC operation.

Since the number of resources used in the TTMMA scheme is about 5.4 times greater than that of the FDMA-based scheme for a single discovery signal, for a fair comparison, UEs are configured to select one of six DRU1s and transmit a discovery signal in the FDMA-based scheme. That is, in the TTMMA scheme, all *n* UEs generate a signal using one DRU2 that includes 912 Res; while in the FDMA-based scheme, *n* UEs select one of the six DRU1s, in which each DRU1 includes 168 REs, through which the signal is transmitted. In the FDMA-based scheme, we assume two cases of resource allocation. One is the ideal resource allocation so that each DRU1 experiences the same level of congestion. Another is randomly selected resource allocation so that each DRU1 experiences different levels of congestion.

Simulations are performed on two different channel models, which are shown in Table 1. The result is based on the average obtained over 50,000 independent experiments. The performance metric is the average number of discovered UEs versus the number of multiple-access UEs. Average number of discovered UEs means average number of discovery signals passed the CRC check per an independent experiment.

**Figure 7:**
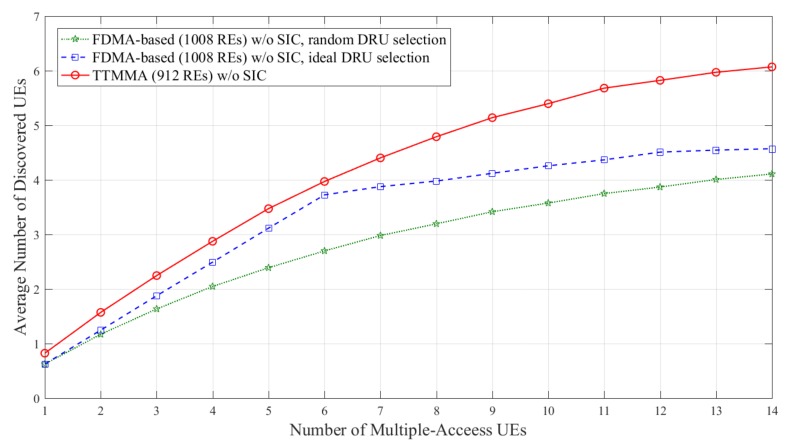
Average number of discovered user equipment (UEs) according to the number of multiple-access UEs in block Rayleigh fading channel without applying successive interface cancellation (SIC).

**Figure 8:**
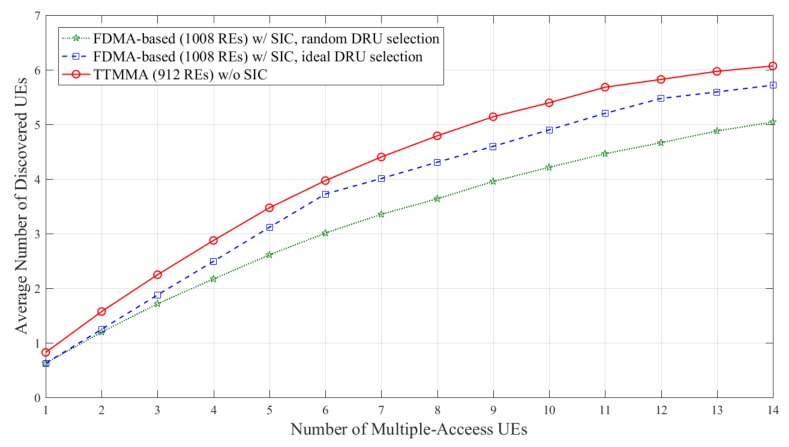
Average number of discovered UEs according to the number of multiple-access UEs in block Rayleigh fading channel, only applying SIC to the frequency division multiple-access (FDMA)-based scheme.

Figure 7 and Figure 8 show the results in block Rayleigh fading channel. In Figure 7, the FDMA-based scheme can discover up to 4.5 UEs without SIC in case of ideal resource allocation, while the TTMMA scheme can discover up to 6 UEs, even though it uses fewer resources and does not perform the SIC.

In Figure 8, the FDMA-based scheme can discover more UEs due to applying the SIC. However, the performance is still less than the TTMMA, even though it uses fewer resources and does not apply the SIC.

**Figure 9:**
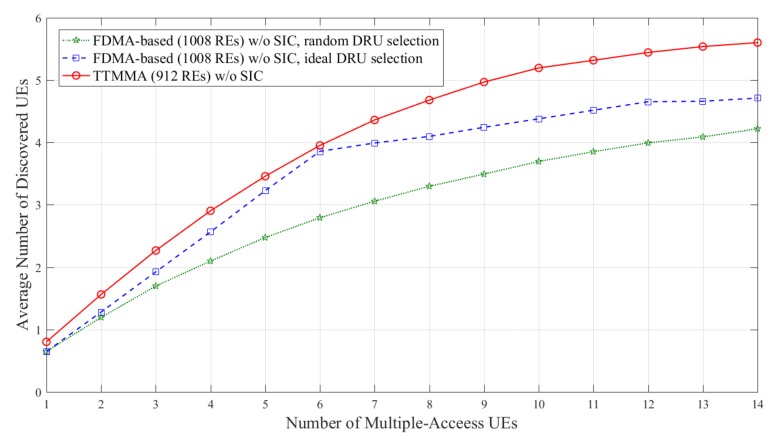
Average number of discovered UEs according to the number of multiple-access UEs in TDL-A channel without applying SIC.

**Figure 10:**
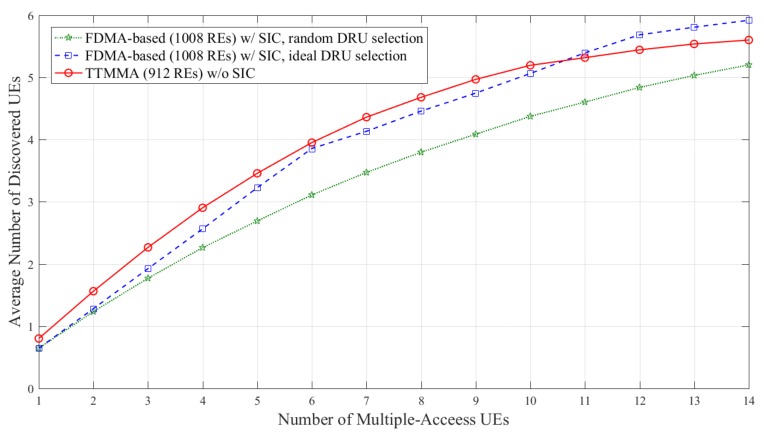
Average number of discovered UEs according to the number of multiple-access UEs in TDL-A channel with only applying SIC to FDMA-based scheme.

Figure 9 and Figure 10 show the results in TDL-A channel. TDL-A channel has frequency selective characteristics. In Figure 9, both FDMA-based and TTMMA experience the performance degradation compared to block Rayleigh fading channel. Although performance degradation in TTMMA is more severe, it shows superior performance compared to the FDMA-based scheme in the case of ideal resource allocation.

In Figure 10, the FDMA-based scheme can discover more UEs due to applying the SIC. In particular, the performance of the FDMA-based scheme is better than that of the TTMMA scheme in cases of more than 11 Multiple-Access UEs. However, the FDMA-based scheme should apply SIC and assume an ideal resource allocation for this performance.

The computational complexities the FDMA-based scheme and TTMMA are compared in terms of the number of additions in Table 2. The decoding in the FDMA-based scheme is composed of the demodulation and polar code successive cancellation list (SCL) decoding [34], but the TTMMA decoding is a sole multi-user demodulation whose complexity is calculated by (12). In Table 2, $N_{modsym}$ is the number of modulated symbols, $N_{constel}$ is the number of constellations, $N_{DRU1}$ is the number of DRU1, L is the list size for SCL decoding, $N_{mc}$ is the mother code size of polar code, and $N_{UE}$ denotes the number of multiple-access UEs. The complexity for SCL decoding of polar codes is evaluated for the simplified LLR-based SCL decoding [35,36]. 

We compare the complexities of both schemes for our performance evaluation scenario. The parameter values for the scenario are given in Table 3. In Table 4, the complexity evaluation is shown for the FDMA-based scheme with or without SIC and for the proposed scheme. In the FDMA-based scheme, the resource region is composed of 6 DRUs. Because knowledge of the number of UEs that share the same resource region is not assumed, a receiver needs to try to decode discovery messages from all DRUs. At least 6 decoding trials are taken in the FDMA-based scheme without SIC. If the scheme runs with SIC, then multiple decoding trials can be carried out for a single DRU. One may repeat the polar code decoding up to the maximum number of times. The minimum number of these trials is 6 since there are 6 orthogonal DRUs, and in our simulation setting the maximum number is 14, the maximum possible number of UEs. The corresponding minimal and maximal complexities are given in Table 4. On the other hand, TTMMA runs with a fixed complexity which is 22% lower than that of the FDMA-based scheme without SIC and the minimal complexity of the SIC scheme. 

## 6. Conclusions

A new multiple-access scheme based on the trellis tone modulation, TTMMA, was proposed. Unlike the conventional FDMA-based scheme, TTMMA performs the discovery of multiple UEs on the same resource. This eliminates the need for strict collision avoidance and collision detection techniques, so that the discovery procedure can be designed concisely. The proposed message-passing demodulation scheme effectively discovers multiple UEs from a single DRU and the discovery capacity is resultantly increased. In addition, the TTMMA scheme generates a single-tone discovery signal to concentrate the signal energy and solve the PAPR problem of OFDM transmission, thereby increasing the transmission signal power and greatly improving the discovery range. The proposed TTMMA scheme significantly outperforms the conventional FDMA-based scheme at lower computational complexity. 

The proposed TTMMA scheme can be used without being limited to the D2D discovery, but to more general multiple-access environments. In particular, the fact that PAPR is set to 1 by transmitting signal generation in a single-tone manner can be a solution to the persistent problem of uplink communication in conventional mobile communication systems. In addition, the capacity of the uplink can be increased by improving the amount of information that can be transmitted with the same resource.

## Figures and Tables

**Table 1:** Evaluation Assumptions.

Parameters	Values
Bandwidth	10 MHz
FFT size	2048
Subcarrier spacing	15 kHz
Transmission power per symbol	23 dBm
Noise power	−174 dBm/Hz
Noise figure	9 dB
Channel model I	Block Rayleigh fading
Channel model II	TDL-A
Delay spread for Channel II	30 ns
Propagation model	D2D outdoor-to-outdoor model
Carrier frequency	2.4 GHz
Height of antenna	1.5 m
Mobile speed for Channel II	5 km/h

**Table 2:** Computational complexity of the FDMA-based discovery and trellis tone modulation multiple-access (TTMMA).

Scheme	Demodulation	Decoding
FDMA-based (w/o SIC)	$N_{modsym}\times N_{constel}\times N_{DRU1}$	$\left\{\begin{array}{c} L\times N_{mc}\times \log_{2}\left(N_{mc}\right)\\ +0.5\times L\times \left(\log_{2}L+1\right)\left(\log_{2}L+2\right) \end{array}\right\}\times N_{DRU1}$
FDMA-based (w/ SIC)	$N_{modsym}\times N_{constel}\times N_{UE}$	$\left\{\begin{array}{c} L\times N_{mc}\times \log_{2}\left(N_{mc}\right)\\ +0.5\times L\times \left(\log_{2}L+1\right)\left(\log_{2}L+2\right) \end{array}\right\}\times N_{UE}$
TTMMA	$\left(N-1\right)\times 2I_{max}\times M\times \left(\overset{d}{\underset{i=1}{\sum }}\left(\begin{array}{c} d\\ i \end{array}\right)\left(i-1\right)+2^{d}\right)$	N/A

**Table 3:** Parameters used in performance evaluation.

$N_{modsym}$	144	$N_{mc}$	512
$N_{constel}$	4	N	75
$N_{DRU1}$	6	$I_{max}$	3
$N_{UE}$	1 ~ 14	M	12
L	8	d	4

**Table 4:** Comparisons of computational complexity in number of additions.

Scheme	Demodulation	Decoding	Total
FDMA-based (w/o SIC)	3456 (for 6 DRUs )	221664 (for 6 DRUs)	225120 (for 6 DRUs)
FDMA-based (w/ SIC)	3456 ~ 8064 (for 14UEs)	221664 ~ 517216 (for 14UEs)	225120 ~ 525280 (for 14UEs)
TTMMA	175824	N/A	175824
